# Impact of different ultrasound-assisted processes for preparation of collagen hydrolysates from Asian bullfrog skin on characteristics and antioxidative properties

**DOI:** 10.1016/j.ultsonch.2022.106163

**Published:** 2022-09-12

**Authors:** Sylvia Indriani, Thanasak Sae-leaw, Soottawat Benjakul, Tran Hong Quan, Supatra Karnjanapratum, Sitthipong Nalinanon

**Affiliations:** a School of Food Industry, King Mongkut’s Institute of Technology Ladkrabang, Ladkrabang, Bangkok 10520, Thailand; b The International Center of Excellence in Seafood Sciences and Innovation, Prince of Songkla University, Songkhla 90110, Thailand; c Faculty of Agro-Industry, Prince of Songkla University, Songkhla 90110, Thailand; d Department of Food Technology, Faculty of Applied Biological Sciences, Vinh Long University of Technology Education, Vinh Long 890000, Vietnam; e Professional Culinary Arts Program, School of Management, Walailak University, Thasala, Nakhon Si Thammarat 80161, Thailand; f Food Technology and Innovation Research Centre of Excellence, Department of Agro Industry, School of Agricultural Technology, Walailak University, Thasala, Nakhon Si Thammarat 80161, Thailand

**Keywords:** UAP, ultrasound-assisted process, UP, UAP as pre-treatment, US, UAP as simultaneous treatment, CH, collagen hydrolysate, ORAC, Oxygen reactive absorbance capacity, FRAP, ferric reducing antioxidant power, FS, fresh skin, Hyp, hydroxyproline, Asian bullfrog skin, Collagen hydrolysate, Ultrasound-assisted processes, Ultrasound pre-treatment, Ultrasound simultaneous treatment, Antioxidative activities

## Abstract

This study focused on impact of ultrasound-assisted process (UAP) at pre-treatment (UP) and simultaneous treatment (US) during papain hydrolysis for preparing collagen hydrolysate (CH) from Asian bullfrog skin. Ultrasonication times were varied (10, 20, 30 min), and CH prepared using papain hydrolysis without UAP was used as control. Different UAPs provided CH with various hydroxyproline contents, α-amino group contents, surface hydrophobicities, and antioxidative activities. UP at 20 min (UP-20) and US at 30 min (US-30) provided highly antioxidative CHs, which were selected for further studies on their Oxygen reactive absorbance capacity (ORAC) and molecular characteristics. CHs from UP-20 and US-30 had higher ORAC than that of control group (*p* ≤ 0.05). Slight difference in amino acid composition was detected between samples. Based on these results, molecular characteristic styles, molecular weight profile, antioxidative peptide content, and secondary structure of each sample were obtained. These results indicate that UP and US used varied enzymatic hydrolysis patterns and modified molecular conformation of CH, resulting in enhanced antioxidative activity. Therefore, different UAPs as UP and US could be effectively used in preparation of CH using papain hydrolysis from Asian bullfrog skin, which could improve production process efficiency by enhancing their bioactivity, particularly antioxidative activity.

## Highlights

• Ultrasound-assisted process was used in frog skin collagen hydrolysate preparation.
• Ultrasound pre-treatment (UP) and simultaneous treatment (US) were studied.
• UP and US at different treatment times provided CH with various characteristics.
• CHs from UP-20 and US-30 used had high antioxidative activities.
• UP and US impacted molecular characteristic and antioxidative activity of CH.

## Graphical abstract

**Figure:**
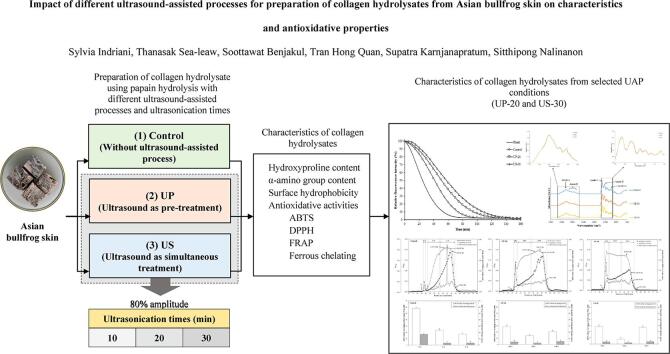

## 1 Introduction

Collagen hydrolysates (CHs) are produced from collagen by acid, alkaline, or enzymatic hydrolysis, resulting in smaller peptides [1]. Several CHs have a high content of essential amino acids that exhibit functional and biological properties [2], and have been produced from various by-products of food and agricultural industries to add value to the supply chain [3], [4]. Enzymatic hydrolysis, a green technology, has been used to prepare several bioactive CHs, in which different proteases are used to enhance hydrolysis and their bioactivities [5]. Papain (EC 3.4.22.2), a protease derived from the latex of papaya (*Carica papaya*), has great potential for the production of antioxidative collagen peptides from various collagenous sources [6]. With the broad specificity (pH 4–10, at temperatures up to 80 °C) of this endopeptidase [7], it could operate practically in the production process with fewer additional chemical and energy costs.

The ultrasound-assisted process (UAP) is another potential green technology because no chemicals are added and it can lower the formation of toxic compounds [8]. Ultrasound has been used to improve protein properties by enhancing enzyme activity, extracting components from materials, and modifying the availability and functional properties of several proteins [9]. Recently, UAP has been used as a pre-treatment (UP) in conjugation with enzymatic hydrolysis to enhance production efficiency and protein characteristics [10], [11], [12]. Furthermore, UAP has been successively used as a simultaneous treatment (US) with enzymatic hydrolysis, in which the combined effect could enhance the enzyme activity and favor the penetration of enzyme into the sample matrix [10], [13]. Nevertheless, Vidal et al. [12] found that simultaneous ultrasound hydrolysis is strongly correlated with the obstruction of enzyme activity during hydrolysis. Consequently, lower production of high-functionality peptides was achieved. Thus, as part of the sufficient energy of ultrasound treatment, the application mode of the UAP in hydrolysis could also be an important parameter that needs to be considered.

In recent decades, bullfrog farming has pitched extensively in the production of emerging food delicacies [14]. This could signify a high quantity of byproducts produced from frog trimming for consumption, where at least 45 % of the frog body is discarded without utilization [15]. Asian bullfrogs and *Rana tigerina*, currently known as *Hoplobatrachus rugulosus*, have been used as alternative protein sources, and the skin of this Asian bullfrog has also been studied as an alternative collagenous source with excellent physicochemical and functional properties [15], [16]. However, there is limited information on CH from this collagenous resource, particularly with UAP. Therefore, the present study aimed to investigate the impact of different UAPs, such as UP and US, using papain hydrolysis, on the production of antioxidative CHs from Asian bullfrog (*R. tigerina*) skin, and the physicochemical and molecular characteristics were also evaluated.

## 2 Materials and methods

### 2.1 Materials

Asian bullfrog skins were procured from a local market in Ladkrabang, Bangkok, Thailand. Papain from *Carica papaya* latex (EC 3.4.22.2; 3,000 U/g), ABTS (2,2′-azino-bis(3-ethylbenzothiazoline-6-sulfonic acid)), DPPH (2,2-diphenyl-1-picryl-hydrazyl-hydrate), iron (II) chloride, iron (III) chloride, TPTZ (2,4,6-Tris(2-pyridyl)-s-triazine), and Trolox were purchased from Sigma–Aldrich (St. Louis, MO, USA). Molecular weight markers were purchased from Fermentas Life Science (#SM0431, USA). All chemicals used in this study were of analytical grade.

### 2.2 Frog skin collection

The skins were kept in a polystyrene box containing ice at a skin/ice ratio of 1:2 (w/w) and transported to the School of Food Industry, King Mongkut’s Institute of Technology, Ladkrabang, Bangkok, within 1 h. Upon arrival, the skins were washed with iced tap water (1–4 °C), packed in polyethylene bags, and stored at –20 ℃ until used. Prior to the CH preparation, the frozen frog skins were cut into small pieces (2.0 × 2.0 cm^2^) using an electric sawing machine (Union Kitchen & Service Co., ltd., Bangkok, Thailand).

### 2.3 Removal of Non-collagenous protein

The skin was removed from non-collagenous compounds following the method described by Karnjanapratum et al. [15] with modifications. Briefly, the prepared skin was soaked in 0.3 M NaOH with a skin/alkaline solution ratio of 1:10 (w/v) for 6 h at 4 °C. The mixture was continuously stirred using an overhead stirrer (model W20.n, IKA®-Werke GmbH & CO.KG, Stanfen, Germany) at 300 rpm, and the fresh alkaline solution was changed every 2-times. Thereafter, the skin was washed using cold tap water (4–5 °C) until the wash water had a slightly basic or neutral pH (7.0–7.5).

### 2.4 Swelling process

Alkaline-treated skins (100 g) were soaked in 1.0 M acetic acid at a skin/solution ratio of 1:10 (w/v). The mixture was stirred at 200 rpm at 4 °C for 4 h. The resulting swollen skin was then washed with cold tap water until neutral or slightly acidic in pH (6.5–7.0) wash water was obtained. The swollen skin was used to prepare CH.

### 2.5 Preparation of CH from Asian bullfrog skin using papain hydrolysis with different UAPs

The swollen skin was subjected to ultrasound-assisted papain hydrolysis under two different modes: ultrasound UP and ultrasound US, with various ultrasonication times (10, 20, and 30 min). These methods were developed and modified as described by Ali et al. [10] and Karnjanapratum and Benjakul [16].

#### 2.5.1 CHs prepared using papain hydrolysis with ultrasound UP

Ultrasound UP was conducted by suspending the swollen skin (50 g) in distilled water at a skin/water ratio of 1:5 (w/v) using UAP (Sonics, Model VC750, Sonica & Materials, Inc., Newtown, USA). A flat-tip probe with a diameter of 25 mm was used with an intensity of 153 W/cm^2^ at a single frequency of 20 kHz with 80 % amplitude. A pulse mode (5 s acting and 5 s resting) was used to avoid overheating during ultrasonication. This UP was performed at various ultrasonication times (10, 20, and 30 min). During the UP, the temperature was maintained at 37–40 °C using a water bath and monitored using a digital thermometer until the end of the process (60 min of total time). The resulting ultrasound -UP skins were subjected to papain hydrolysis.

The ultrasound UP skins were then hydrolyzed using 3 % (w/w) papain and incubated at 40 °C with constant agitation (200 rpm) for 2 h using a water bath shaker (Model Maxturdy-30, DaiHan Scientific Co. ltd., Gangwon-do, Korea) [17]. Papain hydrolysis was terminated by heating at 90 °C for 20 min, and the resulting mixture was centrifuged at 9,000 rpm at 4 °C for 30 min. The supernatant was collected and lyophilized using a freeze-dryer Scanvac CoolSafe 55–4 Pro, Labogene, Denmark). The CHs prepared using ultrasound UP skin at ultrasonication times 10, 20, and 30 min, were referred to as “UP-10,” “UP-20,” and “UP-30,” respectively. The powders were kept in polyethylene bags and stored at −20 °C until use for analysis (within 3 months).

#### 2.5.2 CHs prepared using papain hydrolysis with ultrasound US

The swollen skin was pre-treated without ultrasonication by suspending it in distilled water at a skin/water ratio of 1:5 (w/v) and incubated at 40 °C with constant agitation (200 rpm) for 60 min using a water bath shaker. The pretreated skins were then subjected to papain hydrolysis.

The pretreated skins were then hydrolyzed using 3 % (w/w) papain with ultrasound US (during hydrolysis) at different ultrasonication times (10, 20, and 30 min). This papain hydrolysis with ultrasound US was continued by incubation at 40 °C with constant agitation (200 rpm) for 2 h using a water bath shaker. Thereafter, the reaction was terminated by heating (90 °C, 20 min), centrifugation, and lyophilization, as described previously. The CHs prepared using ultrasound US skin at ultrasonication times 10, 20, and 30 min, were referred to as “US-10,” “US-20,” and “US-30,” respectively. The powders were kept in polyethylene bags and stored at −20 °C until used. The CH prepared using papain hydrolysis from swollen skin without UAP was also evaluated and referred to as “Control”.

### 2.6 Analyses

#### 2.6.1 Hydroxyproline (Hyp) content

Hyp content was determined according to the method described by Bergman and Loxley [18]. The Hyp content was calculated and expressed as mg/g of solid.

#### 2.6.2 α-Amino group content

The α-amino group content was measured according to the method described by Benjakul and Morrisey [19]. The absorbance was measured at 420 nm and a standard curve of l-leucine (0.50 to 5.00 mM) was used. The α-amino group content was expressed as µmol/g solid.

#### 2.6.3 Surface hydrophobicity

The surface hydrophobicity of the CHs was measured according to the method described by Karnjanapratum and Benjakul [16] with a slight modification. The relative fluorescence intensity of the ANS-protein conjugates was measured using a spectrofluorometer (F-2700, Hitachi, Japan) at excitation and emission wavelengths of 374 nm and 485 nm, respectively, at a scanning speed of 5 nm/s. The surface hydrophobicity was calculated from the initial slope of relative fluorescence intensity vs CH concentration (mg/mL) using a linear-regression analysis then the initial slope was referred to as “H_0_ANS”.

#### 2.6.4 Antioxidative activity

ABTS radical scavenging activity.

ABTS (2,2′-azino-bis(3-ethylbenzothiazoline-6-sulfonic acid)) radical scavenging activity was determined using the ABTS assay [20]. Antioxidative activity was expressed as μmol Trolox equivalent (TE)/g sample.

##### 2.6.4.1 DPPH radical scavenging activity

DPPH (2,2-diphenyl-1-picryl-hydrazyl-hydrate) radical scavenging activity was measured according to the method described by Benjakul et al. [21]. Antioxidative activity was expressed as μmol Trolox equivalent (TE)/g sample.

##### 2.6.4.2 Ferric reducing antioxidant power (FRAP)

The FRAP assay was conducted according to the method described by Sae-leaw and Benjakul [22]. Antioxidative activity was expressed as μmol Trolox equivalent (TE)/g sample.

##### 2.6.4.3 Ferrous chelating activity

Ferrous chelating activity was measured according to the method described by Thiansilakul et al. [23]. Antioxidative activity was expressed as μmol EDTA equivalent (EE)/g sample.

### 2.7 Characteristics of selected CHs

CHs prepared using an UAP from each process tested (ultrasound UP and US) with good antioxidative properties (UP-20 and US-30) were selected for further study, where the CH prepared from papain hydrolysis without UAP was also evaluated as the control.

#### 2.7.1 Oxygen reactive absorbance capacity (ORAC)

ORAC was determined according to the method described by Sae-Leaw and Benjakul [22]. The kinetic curve was plotted between the relative fluorescence intensity (%) and time (min), where Trolox was used as a standard. A blank was prepared in the same manner using 75 mM phosphate buffer (pH 7.0) instead of the sample. ORAC was calculated from the area below the curve and expressed as μmol Trolox equivalent (TE)/g sample.

#### 2.7.2 Amino acid composition

The amino acid compositions of the CHs were analyzed as described by Benjakul et al. [24] using an amino acid analyzer (JLC-500/V AminoTac^TM^, JEOL Inc., USA). Amino acid compositions are reported as percentages of the total amino acids.

#### 2.7.3 Protein pattern

Protein patterns were observed by sodium dodecyl sulfate–polyacrylamide gel electrophoresis (SDS-PAGE) as per the method described by Laemmli [25]. The samples were solubilized in 5 % SDS solution (15 µg protein) and then loaded onto polyacrylamide gels (containing 12 % separating gel and 4 % stacking gel). The gels were subjected to electrophoresis using an AE-6440 instrument (20 mA/gel). Molecular weight markers ranging from 14.4 to 116 kDa were used.

#### 2.7.4 Molecular weight (MW) profile

The MW profile of CH was determined using a Sephadex G-100 gel filtration column (1.6 × 60 cm, Sephadex® G-100, 17–0032-01, GE Healthcare Bio-Science AB, Uppsala, Sweden). Diluted samples (50 mg/mL) were prepared using distilled water and filtered through a 0.22 μm polyethersulfone syringe filter membrane (25 mm, id.) (Bioland Scientific LLC, California, USA), and loaded onto the column. Elution was performed using a peristaltic pump (0.5 mL/min, Perista® pump, AC-2110, ATTO Co., Japan) coupled with a fraction collector (Model 2110, Bio-Rad Laboratories Inc., Richmond, CA, USA). Distilled water was used as the eluent, and 3 mL fractions were collected. Absorbance was recorded at 220 and 280 nm. Blue dextran (2,000,000 Da) was used for the void volume measurement. The MW markers used were vitamin B12 (1,355.4 Da), glycine–tyrosine (238.25 Da), potassium dichromate (194.19 Da), and tyrosine (181.2 Da). The MW of the fraction was estimated from the plot between the available partition coefficient (Kav) and the logarithm of the MW of the protein standards. The antioxidative activity of the samples was analyzed using the ABTS radical scavenging activity of all fractions.

#### 2.7.5 Partial purification of antioxidative peptides

The CHs were subjected to partial purification by Sephadex G-100 gel filtration column chromatography, as described above. Based on their MW and ABTS radical scavenging profiles, each CH was fractionated, pooled into three major fractions, and lyophilized. Three major fractions obtained from the control (C-1, C-2, and C-3), UP-20 (UP-1, UP-2, and UP-3), and US-30 (US-1, US-2, and US-3) were analyzed for their ABTS radical scavenging activity and FRAP (as mentioned in Section 2.6.4).

#### 2.7.6 Fourier transform infrared (FTIR) spectra

FTIR spectroscopy of CHs was characterized using attenuated total reflectance FTIR spectrometer model Equinox 55 (Bruker Co., Ettlingen, Germany) equipped with a horizontal ATR through plate crystal cell (45° ZnSe; 80 mm long, 10 mm wide, and 4 mm thick) (PIKE Technology Inc., Madison, WI, USA), as described by Karnjanapratum et al. [15]. The spectral data were analyzed using the OPUS 3.0 data collection software program (Bruker Co., Ettlingen, Germany).

### 2.8 Statistical analysis

A completely randomized design was used in this study. Experiments were performed in triplicate, and the resulting data were subjected to analysis of variance (ANOVA) at a confidence level of 95 % (*p ≤* 0.05). The means were compared using Duncan’s multiple range test. An independent sample *t*-test was performed to compare the means between two samples [26]. Statistical analyses were performed using the Statistical Package for Social Sciences (IBM SPSS Statistics, IBM, New York, USA).

## 3 Results and discussion

### 3.1 Hyp content, α-amino group content, and surface hydrophobicity (H_0_ANS)

UAPs were used as UP and US for papain hydrolysis of Asian bullfrog skin collagen at different ultrasonication times (10, 20, and 30 min). The impact of UAP on the Hyp content of the resulting CH was evaluated, as shown in Table 1. UAP could enhance the Hyp content of the obtained CHs for both UP and US used, compared to that of the control (without UAP) (*p* ≤ 0.05). Increasing the ultrasonication time could increase the Hyp content, particularly in UP, where the highest Hyp content was found in UP-30 (*p* ≤ 0.05). However, a decrease in Hyp content was observed at ultrasonication times longer than 10 min for US. Nevertheless, the Hyp content of the CH prepared using US was higher than that of UP at the same ultrasonication time used, except for those prepared with 30 min ultrasonication time (*p* > 0.05). These results indicated that UAP as a UP provided a loosened skin matrix, where longer ultrasonication resulted in higher dissipated energy. Collagenous materials from the skin can then easily cleft or leach out during papain hydrolysis [16]. Application of UAP as a US in conjugation with papain hydrolysis tended to favor the CH preparation with a higher Hyp content. The combination effect might simplify the penetration and dispersion of papain, while enhancing mass transfer from the skin matrix. In contrast, the co-extractants from frog skin could also be released, which would dilute the Hyp content of CH as found from US when a longer ultrasonication time was used [27]. Therefore, the different UAP used as UP and US determined the Hyp content of CH from Asian bullfrog skin differently. Under sufficient dissipated energy, particularly affected by ultrasonication time, both UP and US could effectively induce the release of Hyp content, which governed the purity of the resulting CH.

**Table 1:** Characteristics of collagen hydrolysates from Asian bullfrog skin prepared using papain hydrolysis with different ultrasound-assisted processes.

Samples	Hydroxyproline content (mg/g solid)	α-amino group content (µmol/g solid)	Surface hydrophobicity (H_0_ANS)
Control	48.19 ± 0.99^e^	730.85 ± 2.70^a^	0.21 ± 0.01^e^
UP-10	51.14 ± 0.87^Bd^	605.59 ± 7.06^Ac^	0.26 ± 0.02^Bd^
UP-20	52.22 ± 0.62^Bd^	644.11 ± 1.53^Ab^	0.31 ± 0.01^Ac^
UP-30	71.48 ± 0.33^Aa^	590.40 ± 16.85^Acd^	0.34 ± 0.01^Bb^
US-10	72.32 ± 2.14^Aa^	602.94 ± 9.05^Acd^	0.35 ± 0.02^Aab^
US-20	62.72 ± 0.26^Ac^	588.17 ± 1.44^Bd^	0.25 ± 0.01^Bd^
US-30	69.38 ± 1.14^Ab^	562.03 ± 11.91^Ae^	0.37 ± 0.01^Aa^

Data are expressed as mean ± standard deviation (n = 3).

Control: Collagen hydrolysates prepared without ultrasound-assisted process.

UP-10, −20, −30: Collagen hydrolysates prepared using ultrasound pre-treatment as assisted process at 10, 20, and 30 min, respectively.

US-10, −20, −30: Collagen hydrolysates prepared using ultrasound simultaneous treatment as assisted process at 10, 20, and 30 min, respectively.

Different uppercase superscripts in the same column indicate significant differences within the same ultrasonication time (*p ≤* 0.05).

Different lowercase superscripts in the same column indicate significant differences (*p* ≤ 0.05).

The α-amino group contents of CHs prepared by UAP under different modes and incubation times are shown in Table 1. Both UP (590.40 – 644.11 μmol/g solid) and US (562.03 – 602.94 μmol/g solid) samples had a significant lower α-amino group content compared to that of control (730.85 μmol/g solid) (p ≤ 0.05). The UP showed an increase in the α-amino group content when the ultrasonication time increased. At the longest treatment time (UP-30), a decline in α-amino group content was observed (*p* ≤ 0.05). Similarly, a lower α-amino group content was found in the US with a longer treatment time (*p* ≤ 0.05). Comparing UP and US, a similar degree of protein cleavage was obtained with the same ultrasonication time. There were only those from the 20 min treatment time, where a higher α-amino group content was attained from UP (*p* ≤ 0.05). An increase in the α-amino group content indicated an increase in the degree of protein hydrolysis and the liberation of small peptides. The loosened skin matrices could be obtained after UP, which resulted in papain cleavage and splitting of peptide bonds in collagen. However, the progressive degradation of collagen via protein unfolding, followed by oxidation or aggregation, forms dense and compact matrices in the skin. This could occur because of the strong and harsh conditions of UAP [16], [28]. The large aggregation could result in a stable and resistant form of skin matrix during papain hydrolysis. This could explain the lower α-amino group content of CH prepared from UP compared to that of the control. However, the use of UAP in combination with enzymatic hydrolysis (US) could cause conformational changes in the enzyme, reducing enzyme activity [13]. The higher intensity of dissipated energy generated from ultrasound under longer ultrasonication time could lead to more severe conformational changes in the enzyme with greater loss of enzyme activity. The results from the present study suggest that UAP as UP and US modes could govern the generation of collagen peptides with different degrees of hydrolysis, which generally determines the characteristics and bioactivity of the resulting CHs.

The H_0_ANS of the CHs prepared using UAP under different modes and ultrasonication times are shown in Table 1. H_0_ANS was used to characterize the tertiary structure of proteins via intramolecular hydrophobic interactions between non-polar amino acid residue side chains, thus affecting protein folding and unfolding [29]. The lowest H_0_ANS was observed in the control (*p* ≤ 0.05), whereas CHs from UAP showed the highest H_0_ANS, regardless of the mode and ultrasonication time used. Considering the same UAP mode, an increase in the ultrasonication time could increase the hydrophobic content of the resulting CHs from UP in a dose-dependent manner (*p* ≤ 0.05). For those from the US, a similar H_0_ANS was obtained for the CHs resulting from different ultrasonication times, except for US-20 (*p* ≤ 0.05). US-20 showed a decrease in hydrophobic content compared with the others. Notably, CHs from US possessed a higher H_0_ANS than those from UP when the ultrasonication time was the same. However, only those treated for 20 min showed a higher hydrophobic content from UP (*p* ≤ 0.05). UAP disrupts the non-covalent bonds of the skin matrices and exposes hydrophobic amino acid residues [30]. Kingwascharapong et al. [31] found that the hydrophobic content of insect proteins could be induced by ultrasound treatment, where a longer ultrasonication time resulted in higher H_0_ANS. Conversely, a decrease in the hydrophobic content of proteins could occur with excessive energy from ultrasound treatment due to protein aggregation via protein–protein interactions [9], [31]. It is well known that optimum peptide length and hydrophobic content can determine the bioavailability of peptides [31], [32]. Therefore, different modes and times of UAP for preparing CH from Asian bullfrog skin in the present study pointed out the crucial impact on the surface properties, especially surface hydrophobicity, which directly determines the functional and biological activities of CH.

### 3.2 Antioxidative activities

**Fig. 1:**
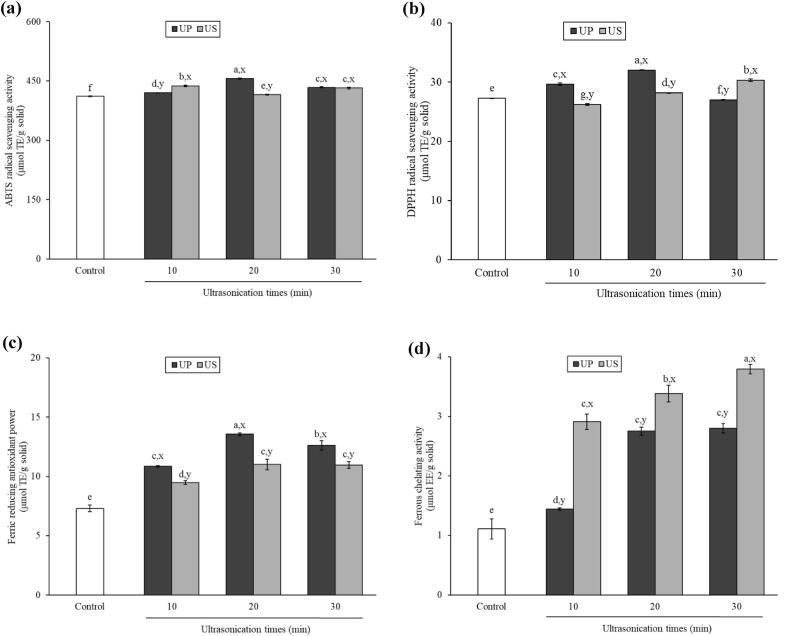
ABTS radical scavenging activity (a), DPPH radical scavenging activity (b), Ferric reducing antioxidant power (c), Ferrous chelating activity (d) of collagen hydrolysates from Asian bullfrog skin prepared using papain hydrolysis with different ultrasound-assisted processes. Bars represent the standard deviation (n = 3). Control: Collagen hydrolysates prepared without ultrasound-assisted process. UP-10, −20, −30: Collagen hydrolysates prepared using ultrasound pre-treatment as assisted process at 10, 20, and 30 min, respectively. US-10, −20, −30: Collagen hydrolysates prepared using ultrasound simultaneous treatment as assisted process at 10, 20, and 30 min, respectively. ^a,b,c,d,…^indicate significant differences between the samples (*p ≤* 0.05). ^x,y^ indicate significant differences between the samples from different modes ultrasound-assisted under the same ultrasonication time (*p ≤* 0.05).

Fig. 1 shows the antioxidative activities of CH prepared from Asian bullfrog skin prepared using UAP under different modes and ultrasonication times, as determined by different assays. ABTS and DPPH radical scavenging assays are based on a single electron transfer mechanism followed by neutralization via electron transfer and/or quenching (hydrogen atom transfer/HAT) [33]. UAP enhanced the ABTS (ABTS) and DPPH radical scavenging activities (DPPH) of the resulting CHs (Fig. 1a and 1b, respectively). For the CHs from UP, ABTS increased with increasing ultrasonication time (Fig. 1a). However, at the longest treatment duration, a decline in ABTS was observed (*p* ≤ 0.05). A similar profile was also found for their DPPH radical scavenging activity, while UP-30 showed a lower DPPH radical scavenging activity than the control (*p* ≤ 0.05) (Fig. 1b). These results indicate that the liberation of antioxidative peptides occurred during papain hydrolysis of Asian bullfrog skin. A higher degree of hydrolysis with low-molecular-weight peptides generally results in higher antioxidative activity [22]. The decrease in the α-amino group content due to protein aggregation induced by the excessive UAP treatment of UP-30 (Table 1) could cause a reduction in its ability to scavenge ABTS and DPPH radicals. A decrease in ABTS was also observed for CH when the UAP was conducted for 20 min (US-20). This relied on a decrease in surface hydrophobicity (Table 1). The ABTS of US-20 increased again with increasing ultrasonication time up to 30 min (US-30), where the hydrophobicity increased with decreasing α-amino group content (Table 1). This result suggests the impact of surface properties, particularly surface hydrophobicity, on the antioxidative activity of peptides, in which US could cause protein unfolding and expose its hydrophobic region on the surface [34]. However, the CHs from US revealed a gradual increase in DPPH radical scavenging activity with an increase in the α-amino group content but varying hydrophobicity. These results suggest that, apart from size and surface properties, the configuration of proteins, including the shape of the peptide, might also govern its antioxidative activity [31]. It was previously reported that different UAP conditions result in variations in protein configuration, depending on the amino acid composition of the peptides [35].

The FRAP and ferrous chelating activity of the resulting CHs were also investigated, as shown in Fig. 1c and 1d, respectively. Different modes and ultrasonication times of UAP used in the present study could significantly enhance the ability of CHs to reduce and chelate metal agents by increasing their FRAP and ferrous chelating activity compared with those of the control (*p* ≤ 0.05). For FRAP of CHs from UP, a similar profile was observed compared with ABTS and DPPH (Fig. 1a and 1b), where the decrease in FRAP occurred when the ultrasonication time was high (30 min). Nevertheless, the CH from US showed an improved FRAP with an increased ultrasonication time, and the highest activity could be reached at ultrasonication time longer than 10 min (*p* ≤ 0.05). It was also found that the ferric chelating activity of UP, in which ultrasound UP for 20 min could be sufficient for preparing antioxidative peptides that function as prooxidants. Conversely, a longer ultrasound US time significantly increased the ferric chelating activity of CHs from the US, where the highest activity was obtained from US-30 (*p* ≤ 0.05). Based on these results, the different UAP modes and ultrasonication times used for preparing CH from Asian bullfrog skin with papain hydrolysis produced different peptides, leading to different antioxidative activities.

Above all, CHs from both UP and US under different treatment times could function as good radical scavengers, electron donors, and metal chelators. UAP as UP at 20 min (UP-20) and US at 30 min (US-30) provided CHs with high antioxidative activity. Thus, these UAP conditions were selected for preparing CH from Asian bullfrog skin using papain hydrolysis and subjected to further study in comparison with that of the control (without UAP).

### 3.3 Characteristics of CHs prepared using selected UAP

#### 3.3.1 Oxygen radical absorbance capacity (ORAC)

**Fig. 2:**
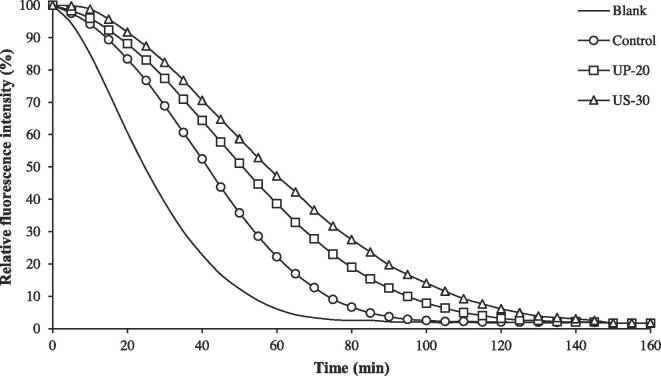
Oxygen reactive absorbance capacity (ORAC) at different times of collagen hydrolysates from Asian bullfrog skin prepared using papain hydrolysis with selected ultrasound-assisted processes. Blank: Distilled water. Control: Collagen hydrolysates prepared without ultrasound-assisted process. UP-20: Collagen hydrolysates prepared using ultrasound pre-treatment as assisted process at 20 min. US-30: Collagen hydrolysates prepared using ultrasound simultaneous treatment as assisted process at 30 min.

The ORAC of CHs prepared using the selected UAP (UP-20 and US-30) was reported as the fluorescence decay at different times in Fig. 2, in comparison with that of the control. Antioxidative capacity was quantified based on the net integrated area under the fluorescence decay curve (AUC), where the blank represents the change in fluorescence intensity of the system without sample addition. ORAC values of control, UP-20, and US-30, were calculated and expressed as Trolox equivalents at 19.46, 33.41, and 42.84 mmol TE/100 g solid, respectively. This result signified that the use of ultrasound could enhance the peroxyl radical scavenging of CH, in which a stronger capacity could be observed from US-30 (*p* ≤ 0.05). The Hyp content of US-30 was higher than that for UP-20 and control (*p* ≤ 0.05) (Table 1). US-30 had the lowest α-amino group content, with the highest surface hydrophobicity, followed by UP-20 (*p* ≤ 0.05). This result indicated that the small peptides with the highest hydrolysis degree in the control with less hydrophobicity could not influence ORAC as much as UAP did. A possible reason for this result is the different peptide conformations. The bioactive peptides were liberated from Asian bullfrog skin during papain hydrolysis with US, and these peptides could be further treated by ultrasonic waves, resulting in the unfolding of peptides with a highly hydrophobic surface [12], [36]. A lower degree of ultrasound modification could also occur in the UP. The ultrasound UP skin could enhance the collagenous skin matrix change before cleavage and release the bioactive peptide in the hydrolysis step. This caused different skin matrices to be used in collagen hydrolysis with UP and US, resulting in different patterns of peptide cleavage by papain. Therefore, UP-20 and US-30 could induce peroxyl radical removal of the resulting CHs differently, and different molecular characteristics could cause this variation and need to be clarified.

#### 3.3.2 Amino acid composition

Table 2 shows the amino acid composition of CHs prepared using papain hydrolysis from Asian bullfrog skin with selected UAP (UP-20 and US-30) in comparison with the control. Six major amino acids were found in all samples as follows: glycine (Gly:16.09–17.12 %), proline (Pro:15.31–15.86 %), arginine (Arg:11.28–12.22 %), hydroxyproline (Hyp:9.72–10.00 %), glutamic acid (Glu:9.28–9.94 %), and alanine (Ala:8.32–8.67 %). All samples exhibited a similar amino acid composition to collagen extracted from Asian bullfrog skin [37]. Gly is the predominant amino acid in collagen, contributing to intramolecular hydrogen bonding perpendicular to the helical axis, thus stabilizing its structure [38]. All tested samples contained significant amounts of hydrophobic amino acids, including Ala, Leu, Met, Phe, Pro, Thr, and Val, which have been reported in antioxidative peptides [11], [24]. Previous studies have reported that the higher hydrophobic amino acids in proteins and their hydrolysates govern the higher antioxidative activities [24], [31]. Based on the amino acid composition in the present study, the control had the highest hydrophobic amino acid content (9.76 %), followed by UP-20 (9.26 %) and US-30 (9.11 %), which was not in accordance with their antioxidative activities. The antioxidative properties of peptides are known to be greatly influenced not only by their amino acid composition or peptide size, but also by the molecular configuration that determines peptide folding behavior and exposure of hydrophobic patches on their surface [11], [34], [39]. The results indicated that the different amino acid compositions of CHs might govern changes in the secondary structure of peptides induced by UAP, which was more likely related to the variation in their antioxidative activity [40].

**Table 2:** Amino acid composition of collagen hydrolysates from Asian bullfrog skin prepared using papain hydrolysis with selected ultrasound-assisted processes.

Amino acids	Content (% of total amino acid)		
	Control	UP-20	US-30
Aspartic acid (Asp)	5.79	5.60	5.56
Cysteine (Cys)	ND	ND	ND
Glutamic acid (Glu)	9.28	9.67	9.94
Glycine (Gly)	16.09	16.73	17.13
Histidine (His)	ND	ND	ND
Hydroxylysine (Hyl)	1.26	1.14	1.17
Hydroxyproline (Hyp)	9.72	9.80	10.00
Isoleucine (Ile)	ND	ND	ND
Alanine (Ala)	8.67	8.53	8.32
Arginine (Arg)	11.28	12.22	11.91
Leucine (Leu)	3.31	3.00	2.96
Lysine (Lys)	4.09	4.07	4.23
Methionine (Met)	1.53	1.42	1.29
Phenylalanine (Phe)	2.51	2.41	2.31
Proline (Pro)	15.86	15.42	15.31
Serine (Ser)	4.41	4.28	4.21
Threonine (Thr)	2.27	2.17	2.22
Tryptophan (Trp)	0.28	0.14	0.16
Tyrosine (Tyr)	1.35	1.22	1.12
Valine (Val)	2.30	2.17	2.15
Imino acids (Hyp + Pro)	25.58	25.22	25.31

ND: not detectable.

Control: Collagen hydrolysates prepared without ultrasound-assisted process.

UP-20: Collagen hydrolysates prepared using ultrasound pre-treatment as assisted process at 20 min.

US-30: Collagen hydrolysates prepared using ultrasound simultaneous treatment as assisted process at 30 min.

#### 3.3.3 MW distribution of CHs and their antioxidative peptides

**Fig. 3:**
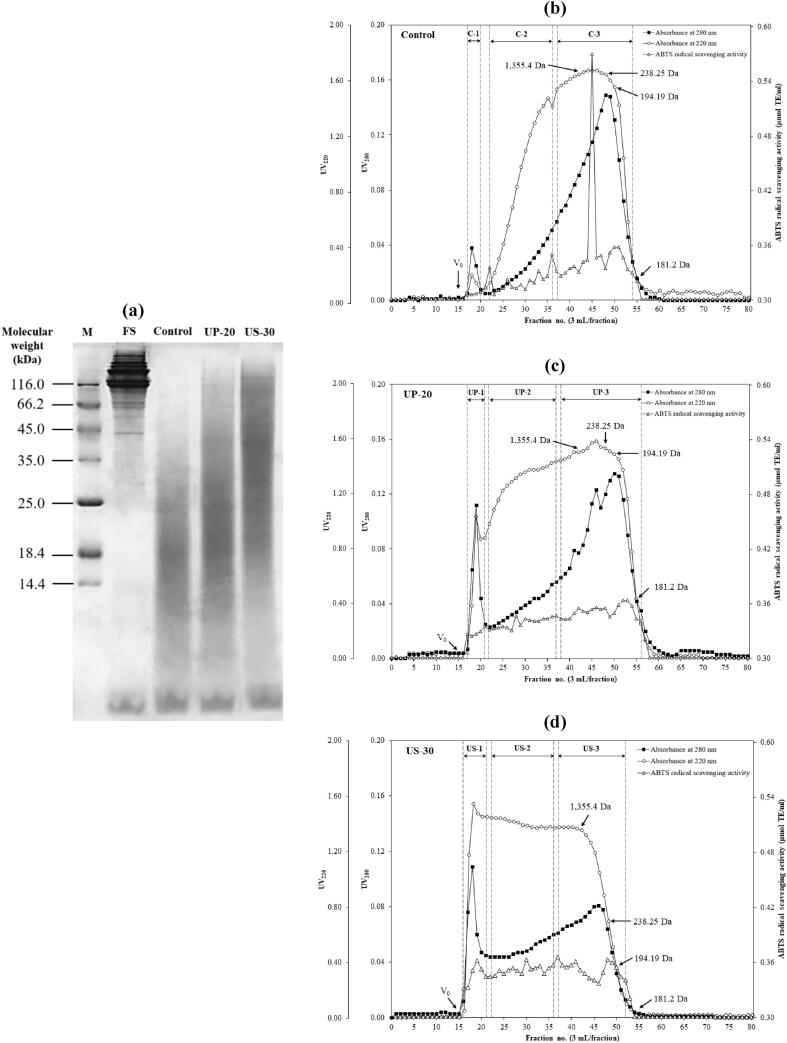
Molecular weight profile by SDS-PAGE (a) and elution profile size exclusion chromatography with Sephadex G-100 (b, c, d) of collagen hydrolysates from Asian bullfrog skin prepared using papain hydrolysis with selected ultrasound-assisted processes. M: Protein markers. FS: Fresh skin. Control: Collagen hydrolysates prepared without ultrasound-assisted process. UP-20: Collagen hydrolysates prepared using ultrasound pre-treatment as assisted process at 20 min. US-30: Collagen hydrolysates prepared using ultrasound simultaneous treatment as assisted process at 30 min.

The MW distribution of Asian bullfrog skin CHs prepared using selected UAP (UP-20 and US-30) was evaluated by SDS-PAGE and size exclusion chromatography, as shown in Fig. 3, in comparison with those of the control. Based on the SDS-PAGE results (Fig. 3a), fresh skin (FS) of Asian bullfrogs showed MW bands similar to that of its type I collagen consisting of β- and γ-chains (175–250 kDa) as well as α_1_- and α_2_-chains (79–175 kDa), as reported by Indriani et al. [37]. Protein hydrolysis through papain cleavage of the native collagen in FS was detected as smeared bands of CH on the gel with the degradation of α-, β-, and γ-chains to smaller peptides. None of the samples had high MW peptides after papain hydrolysis, either with or without UAP (Fig. 3a). The smear band of the control was mainly located at the MW ranging from 10 to 25 kDa, which was lower than those of UP-20 (15–30 kDa) and US-30 (20–110 kDa). This coincided with the free α-amino group contents of the samples (Table 1). Furthermore, the MW distribution of the samples was confirmed using Sephadex G-100 gel filtration chromatography, and the absorbance at 280 nm (UV_280_) and 220 nm (UV_220_) were monitored together with ABTS radical scavenging activity (Fig. 3b–3d). Peptides with aromatic side chain groups were detected by UV_280_ and the presence of peptide bonds was monitored by UV_220_. Different elution profiles were obtained for each of the samples. The control (Fig. 3b) had a greater amount of low-MW peptides containing aromatic amino acids (UV_280_) than UP-20 (Fig. 3c) and US-30 (Fig. 3d). Based on the representative peptides (UV_220_), US-30 contained a larger peptide with the highest amount among others, while the distinctive lower amount of the large molecule was observed in the control (Fig. 3b). This result was in accordance with their MW distribution on the SDS-PAGE gel (Fig. 3a) and the α-amino group content (Table 1). The control fraction with a MW of 885 Da (Fig. 3b) exhibited the highest ABTS radical scavenging activity, followed by the fractions with MW of 196–238 Da. No predominant fraction possessed ABTS radical-scavenging activity for UP-20 (Fig. 3c) and US-30 (Fig. 3d). Nevertheless, every fraction showed the ability to donate hydrogen atoms to some degree, especially US-30. In general, small peptides exhibit high antioxidant activities. However, this also depends on the amino acid composition, sequence, shape, and charge of peptides, as well as molecular conformation [36], [41]. With different UAP modes used (UP-20 and US-30), various hydrolysis patterns occurred, and different MW profiles and antioxidative peptide contents were obtained.

**Fig. 4:**
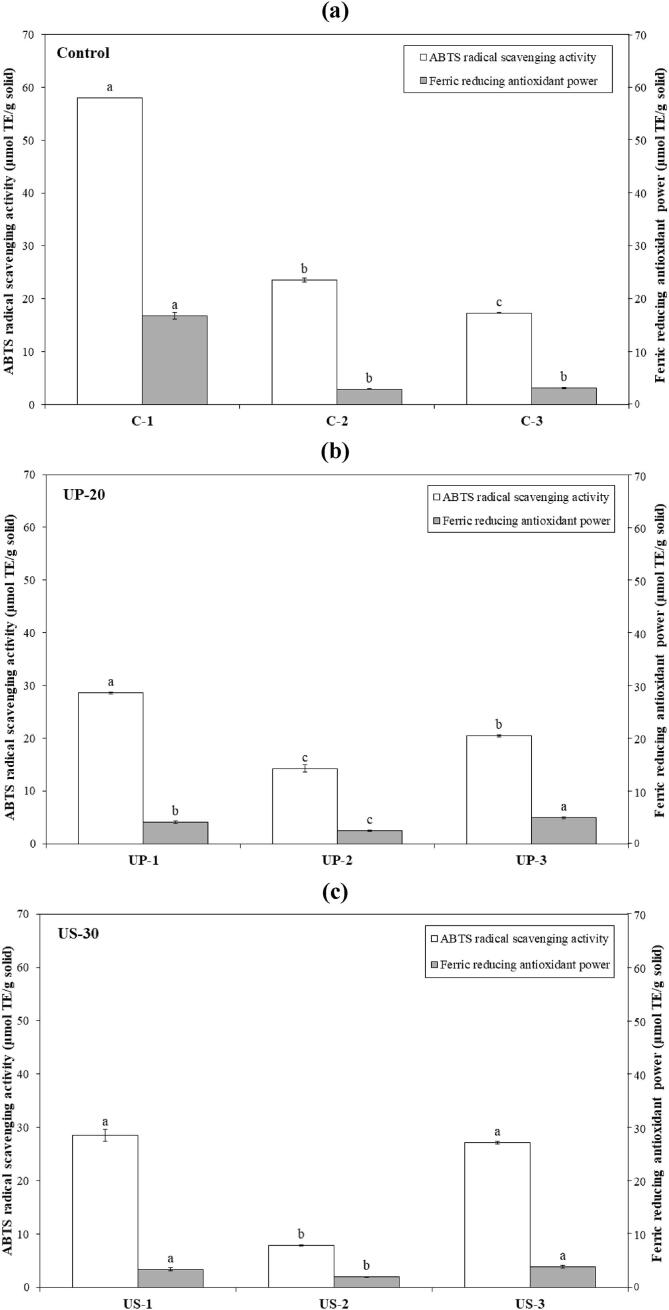
ABTS radical scavenging activity and Ferric reducing antioxidant power of partial purified peptide from collagen hydrolysates of Asian bullfrog skin prepared using papain hydrolysis with selected ultrasound-assisted processes. Control: Collagen hydrolysates prepared without ultrasound-assisted process. UP-20: Collagen hydrolysates prepared using ultrasound pre-treatment as assisted process at 20 min. US-30: Collagen hydrolysates prepared using ultrasound simultaneous treatment as assisted process at 30 min. Bars represent the standard deviation (n = 3). Different letters on the bars indicate significant differences between the samples under the same assay (*p ≤* 0.05).

To identify the antioxidative peptides of CHs from Asian bullfrog skin prepared using selected UAP, each sample was then partially purified, in which the three different regions were derived based on their elution profiles (Fig. 3b–3d) and pooled into three fractions. The antioxidant activity of the pooled fractions from each sample was evaluated using ABTS and FRAP assays (Fig. 4). For the control sample, fraction C-1 showed the highest antioxidative activity for both ABTS and FRAP among the fractions at 57.95 and 16.76 μmol TE/g solid, respectively (Fig. 4a). This result was not relevant to the ABTS profile (Fig. 3b), where the major antioxidative peptide was found in the low-MW region (C-3). This might indicate a dilution effect after pooling all fractions of that region, resulting in a lower ABTS in C-3. Considering the fraction yield (data not shown), the main antioxidative peptide from the control was C-1, yielding only 1.97 % yield. The major component covering almost 50 % of CH (48.64 %) was obtained from C-3, which showed the lowest antioxidant peptide content of the control. This indicated the less antioxidative activity of control, compared with others (Fig. 1, Fig. 2). This hypothesis could also explain the higher antioxidant activity observed in UP-20. The largest (UP-1) and lowest (UP-3) MW fractions possessed predominant antioxidative peptide content in UP-2, contributing 40.43 % of the CH. A similar antioxidative profile was observed for US-30 (Fig. 4c); however, the main antioxidative peptides (US-1 and US-3) contributed only 33.32 %. The fraction with the lowest antioxidative activity, US-2, was the major component in US-30, showing the highest activity among the CHs, particularly ORAC (Fig. 2). This could be explained by the synergistic effect of each antioxidative peptide [42]. Mixtures of those fractions (US-1, US-2, and US-3) as naturally occurred in US-30 could urge the cooperating action for bioactivity enhancement, particularly antioxidative activity. These results verified that a low MW was not the only factor influencing the antioxidative properties of CH. The UAPs used not only determined the papain hydrolysis pattern, but also induced changes in the molecular structure, resulting in enhanced antioxidative properties. Overall, UP-20 and US-30 used for preparing CH with enzymatic hydrolysis in the present study could improve their antioxidative activity by generating various antioxidative peptide contents.

#### 3.3.4 FTIR spectra

**Fig. 5:**
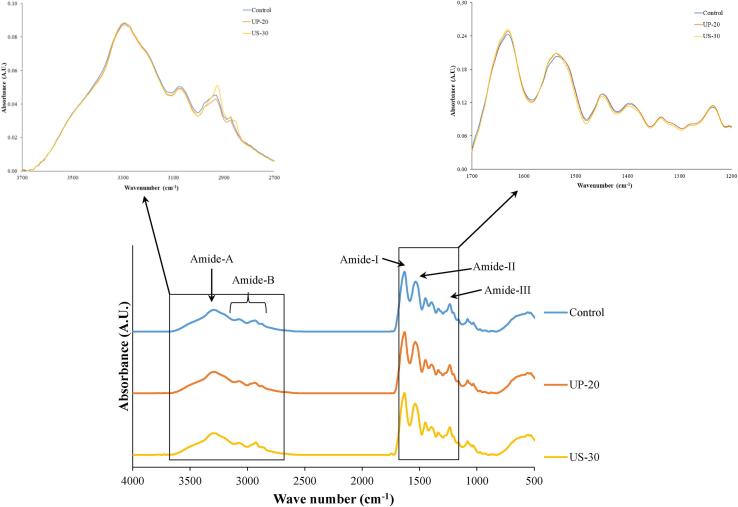
FTIR spectra of collagen hydrolysates from Asian bullfrog skin prepared using papain hydrolysis with selected ultrasound-assisted processes. Control: Collagen hydrolysates prepared without ultrasound-assisted process. UP-20: Collagen hydrolysates prepared using ultrasound pre-treatment as assisted process at 20 min. US-30: Collagen hydrolysates prepared using ultrasound simultaneous treatment as assisted process at 30 min.

The FTIR spectra of CHs from Asian bullfrog skin prepared using the selected UAP conditions, UP-20 and US-30, are presented in Fig. 5 in comparison with the control. All samples showed characteristic absorption peaks in the regions of amides A, B, I, II, and III as the dominant bands. Amide A corresponds to the N—H stretching vibrations when the NH group of the peptide is involved in hydrogen bonding. Amide B contributes to the asymmetric stretching of = C—H and NH_3_^+^ [43]. A similar wavenumber of the amide A band between UP-20 and US-30 (3294 cm^−1^) was observed, which was lower than that of the control (3292 cm^−1^). In addition, a lower band intensity was observed for UP-20 than for the other samples. Lower wavenumbers of the amide B band were observed in the treated samples (UP-20 and US-30), where US-30 showed the highest amplitude, followed by UP-20 and control samples. These shifts in wavenumbers of UAP-treated samples and the lower peak intensity suggested protein–protein interactions via hydrogen bond formation [28], [36]. This might postulate protein aggregation induced by UAP, and a molecular interaction with a more compact structure of peptide molecules could be obtained from UP-20 than from US-30. The amide I band indicated the stretching vibration of the peptide carbonyl group (–C=O), where all samples possessed amide I (1631 cm^−1^) at the same position with different peak intensities. CH prepared from US-30 had the highest amplitude, followed by UP-20 and the control. This result suggests a higher degree of C=O group expression, where protein unfolding with inter-molecular cross-links might be destroyed and folded because of UAP treatment [16]. The amide II peak is attributed to N—H bending coupled with C—N stretching within the peptides [44]. Amide II of the UAP-treated samples was found at the same position (1538 cm^−1^), while a slightly lower wavenumber (1537 cm^−1^) with a lower amplitude was observed in the control. This could imply that a greater proportion of N—H in the control was involved in hydrogen bonding between adjacent chains, compared with others. Amide III corresponds to the combination of N—H deformation and C—N elongation, as well as the wagging vibration of the CH_2_ groups [44]. The peak of the amide III region was located at the same wavenumber (1236 cm^−1^) for all samples, and the highest amplitude was found for UP-20, followed by US-30 and the control. This indicated a higher proportion of the ordered structure of UP-20 with a lower degree of random coil structure. This could reconfirm the protein–protein interaction via protein aggregation of the ultrasound UP-20 used, where the less packed structure could be observed from ultrasound US-30. Therefore, the selected UAP (UP-20 and US-30) used for preparing CH from Asian bullfrog skin obviously impacted the secondary structure and functional groups of CHs, which resulted in the improvement of their antioxidative properties.

## 4 Conclusion

CH was successfully prepared from Asian bullfrog skin using papain hydrolysis with the aid of UAP as a UP and US at different ultrasonication times. The variations in Hyp content, α-amino group content, and surface hydrophobicity of CHs were due to the different UAPs used. The antioxidative activity of CH was successfully enhanced by UAP in both the UP and US. UP-20 and US-30, which have high antioxidative activities, were selected for further study of their amino acid composition, MW distribution, and molecular characteristics. UP-20 and US-30 showed a slight difference in amino acid composition with a distinct MW profile, antioxidative peptide content, and secondary structure. Therefore, the application of UAP as UP and US for preparing CH from Asian bullfrog skin using papain hydrolysis could be considered as an alternative technique to enhance production efficiency by improving its bioactivity, particularly antioxidative activity.

## Declaration of Competing Interest

The authors declare that they have no known competing financial interests or personal relationships that could have appeared to influence the work reported in this paper.

## Data Availability

No data was used for the research described in the article.
